# Einsatz von Impfstoffen zur Prävention bakterieller Resistenzentwicklung

**DOI:** 10.1007/s00103-026-04228-4

**Published:** 2026-04-07

**Authors:** Alexander Klimka, Liza Marie Maus

**Affiliations:** 1 Universität zu Köln, Medizinische Fakultät und Uniklinik Köln, Institut für Medizinische Mikrobiologie, Immunologie und Hygiene, Robert-Koch-Str. 21, 50931 Köln, Deutschland; 2 Deutsches Zentrum für Infektionsforschung (DZIF), Partnerstelle Bonn-Köln, Köln, Deutschland

**Keywords:** Aktive Immunisierung, Passive Immunisierung, Antimikrobielle Resistenz, Adjuvans, Vakzine, Active immunization, Passive immunization, Antimicrobial resistance, Adjuvant, Vaccine

## Abstract

Impfungen gegen bakterielle Erreger spielen eine wichtige Rolle in der Prävention von Infektionen, insbesondere angesichts der wachsenden Antibiotikaresistenz. Historisch markierten Impfstoffe gegen Cholera, Diphtherie und Pertussis (Keuchhusten) erste bedeutende Fortschritte im Kampf gegen bakterielle Krankheiten. Später kamen Impfstoffe unter anderem gegen Meningokokken und Tetanus dazu. Bei diesen aktiven Immunisierungen wird das Immunsystem mit Teilen des Erregers konfrontiert, was die Bildung von Antikörpern, T‑Helferzellen, B‑Zellen und Gedächtniszellen anregt und dadurch einen langfristigen Schutz entstehen lässt. Die gezielte Entwicklung neuer Impfstoffe, auch gegen schwer behandelbare bakterielle Erreger, wird durch eine große Bandbreite von verschiedenen Impfstoffarten ermöglicht, darunter inaktivierte, toxoidbasierte und rekombinante Impfstoffe, sowie moderne Ansätze, wie die reverse Vakzinologie und messenger Ribonucleic Acid (mRNA)-Technologien verbunden mit verstärkenden Adjuvanzien. Neben der aktiven Immunisierung spielt die passive Immunisierung eine wichtige Rolle, bei der erregerspezifische Antikörper direkt verabreicht werden.

Auf Bevölkerungsebene zeigen sich durch Impfprogramme deutliche epidemiologische Effekte, da Impfungen nicht nur Einzelpersonen, sondern auch Gemeinschaften schützen und damit zur Volksgesundheit beitragen. Trotz klarer medizinischer und ökonomischer Vorteile von Impfprogrammen muss deren Akzeptanz in der Bevölkerung durch objektive Informationspolitik noch deutlich erhöht werden. Derzeit befinden sich zahlreiche Impfstoffe in der Entwicklung, die durch innovative Technologien neue Wege eröffnen, bakterielle Infektionen wirksam zu verhindern und zu kontrollieren – ein entscheidender Schritt in einer Ära zunehmender Antibiotikaresistenz.

## Abstract (en)

Vaccinations against bacterial pathogens play a crucial role in infection prevention, especially given the growing prevalence of antibiotic resistance. Historically, vaccines against cholera, diphtheria, and pertussis (whooping cough) marked the first significant advances in the fight against bacterial diseases. Later, vaccines against meningococcus and tetanus, among others, were added. These active immunizations expose the immune system to components of the pathogen, stimulating the production of antibodies, T helper cells, B cells, and memory cells, thereby providing long-term protection. The targeted development of new vaccines, even against difficult-to-treat bacterial pathogens, is enabled by a wide range of vaccine types, including inactivated, toxoid-based, and recombinant vaccines, as well as modern approaches such as reverse vaccinology and messenger Ribonucleic Acid (mRNA) technologies combined with enhancing adjuvants. In addition to active immunization, passive immunization, in which pathogen-specific antibodies are administered directly, also plays an important role.

At the population level, clear epidemiological effects of vaccination programs are evident, as vaccinations protect not only individuals but also communities, thus contributing to public health. Despite the clear medical and economic advantages of vaccination programs, their acceptance among the population must be significantly increased through objective information policies. Currently, numerous vaccines are under development, which, through innovative technologies, are opening up new avenues for effectively preventing and controlling bacterial infections—a crucial step in an era of increasing antibiotic resistance.

## Einleitung

Antibiotika gehören zu den bedeutendsten medizinischen Errungenschaften des 20. Jahrhunderts und haben seit ihrer Einführung Millionen von Menschenleben gerettet. Sie ermöglichen die effektive Behandlung zahlreicher bakterieller Infektionen, die vor ihrer Verfügbarkeit oftmals tödlich verliefen [1]. In den letzten Jahrzehnten zeigt sich jedoch, dass diese Errungenschaft durch Herausforderungen zunehmend eingeschränkt wird: Vor allem die Häufigkeit von Bakterien, die gegen eine oder mehrere Antibiotikaklassen resistent sind, steigt weltweit an [2]. Zusätzlich gehen einige Antibiotika mit starken Nebenwirkungen einher, die ebenfalls beachtet werden müssen. Diese Entwicklung stellt eine erhebliche Gefahr für die moderne Medizin dar, da viele etablierte Therapien, darunter chirurgische Eingriffe, Krebstherapien oder Organtransplantationen, auf eine zuverlässige Infektionskontrolle angewiesen sind. 2016 wurde im sogenannten Jim O’Neill Report prognostiziert, dass ab 2050 jährlich weltweit 10 Mio. Menschen an Infektionen durch antibiotikaresistente Bakterien sterben werden [3]. Eine neuere Analyse von Daten aus den Jahren 1990 bis 2021 erwartet allerdings, selbst unter optimistischen Annahmen, ab 2050 jährlich 28 Mio. Todesfälle durch antimikrobielle Resistenzen (AMR; [4]).

Die Ursachen für die zunehmende Resistenzentwicklung sind vielfältig. Neben der Über- und Fehlverschreibung von Antibiotika in der Humanmedizin tragen unvollständige Therapien, unkontrollierte Selbstmedikationen sowie der umfangreiche Einsatz antimikrobieller Substanzen in der Tierhaltung maßgeblich zur Selektion resistenter Stämme bei [2, 5]. Die Entwicklung neuer Antibiotika ist hingegen seit über 40 Jahren stark rückläufig und bereits kurz nach der Einführung neuer Antibiotika wurden bzw. werden resistente Stämme beschrieben. Insbesondere gegen gramnegative Erreger wurden keine neuen Wirkmechanismen in die Klinik eingeführt. Auch wenn sich derzeit neuartige antimikrobielle Wirkstoffe in der klinischen Entwicklung befinden [6], ist das therapeutische Arsenal momentan erheblich eingeschränkt. Obwohl Hygieneverbesserungen und gezielte Antibiotikaverschreibung (Antibiotic Stewardship) wichtige Bestandteile der AMR-Bekämpfung darstellen, reichen diese Maßnahmen allein nicht aus, um den Trend umzukehren [7].

Vor diesem Hintergrund gewinnt die Prävention von Infektionen zunehmend an Bedeutung. Impfstoffe nehmen hierbei eine Schlüsselrolle ein, da sie durch die Verhinderung primärer Infektionen nicht nur die Krankheitslast senken, sondern auch indirekt den Einsatz von Antibiotika und damit den Selektionsdruck auf Bakterienpopulationen reduzieren [8, 9]. Die doppelt präventive Wirkung – Schutz des Individuums und Reduktion der Transmission – macht sie zu einer der effektivsten Maßnahmen im Kampf gegen AMR. In diesem Zusammenhang ist auch die Gabe von Antikörpern gegen bakterielle Pathogene zu nennen (passive Immunisierung), die eine Alternative zu Antibiotika in der Therapie von bakteriellen Infektionen darstellt. In den letzten 40 Jahren wurden, dank vieler revolutionärer Techniken, nicht nur zahlreiche Impfstoffe zugelassen, sondern es stehen auch Impfstoffe gegen viele resistente Bakterienstämme für die weitere Entwicklung bereit, wie in diesem Artikel dargestellt wird. Zudem wird ein Überblick über die historische Entwicklung von Impfstoffen gegeben und die Wirkmechanismen der aktiven und passiven Immunisierung werden erklärt. Die epidemiologischen Effekte von Impfungen gegen bakterielle Infektionen sowie die ökonomischen und gesundheitspolitischen Effekte von Impfprogrammen werden ebenfalls betrachtet.

## Historische Entwicklung von Impfungen

Die Geschichte der Impfung reicht zurück bis ins späte 18. Jahrhundert, als Edward Jenner 1796 erstmals erfolgreich das Kuhpockenvirus (Orthopoxvirus vaccinia) zur Immunisierung gegen die deutlich gefährlicheren echten Pocken (Orthopoxvirus variolae) einsetzte [10]. Dies markierte einen Wendepunkt in der Medizingeschichte. Bereits zuvor war mit der sogenannten Variolation, also der Verabreichung von Pustelinhalt oder getrocknetem Schorf von echten Pocken, versucht worden, eine Immunität gegen die Pocken zu induzieren. Dies war jedoch aufgrund einer signifikanten Sterblichkeit von 2–3 % ein riskantes Verfahren. Die Entdeckung Jenners führte schließlich zum Begriff „Vaccine“, abgeleitet vom lateinischen „vacca“ für Kuh. Louis Pasteur entwickelte 80 Jahre später die zweite Impfung, indem er einen Speichelextrakt eines tollwütigen Hundes in Hasen injizierte und aus diesen nach einigen Tagen die Wirbelsäule präparierte und trocknete. Aus diesem Präparat wurde so ein Impfstoff extrahiert, den er Tollwutpatienten zum Aufbau einer Immunität vor Ausbruch der Krankheit verabreichte, ohne den durch die Trocknung abgeschwächten Virus als Erreger zu kennen [11].

Auch gegen bakterielle Erreger wurden im Laufe des 19. und 20. Jahrhunderts bedeutende Impfstoffe entwickelt. Beispiele sind die Einführung des Diphtherietoxoid-Impfstoffs in den 1920er-Jahren, der Tetanustoxoid-Impfung in den 1940er-Jahren und des Pertussis-Ganzkeimimpfstoffs, der später durch azelluläre Präparate mit detoxifiziertem Pertussistoxin ersetzt wurde [12]. Ein weiterer Meilenstein war die Zulassung von Polysaccharid-Konjugatimpfstoffen gegen *Haemophilus influenzae* Typ b (Hib) in den 1990er-Jahren, die in vielen Ländern zur nahezu vollständigen Eliminierung invasiver Hib-Erkrankungen geführt hat [13]. Dies sind Beispiele einer aktiven Immunisierung, die im Gegensatz zu einer passiven Immunisierung (Verabreichung von spezifischen Antikörpern gegen den Erreger) zu einer langlebigen Immunität führt.

## Aktive Immunisierung

Impfstoffe zur aktiven Immunisierung gegen bakterielle Erreger werden aktuell in 4 verschiedene Arten klassifiziert: 1. inaktivierte Ganzkeimimpfstoffe (Whole Cell Antigen, WCA), 2. lebend attenuierte Impfstoffe (Live Attenuated Vaccines, LAV), 3. Toxoidimpfstoffe sowie 4. Polysaccharid-Konjugatimpfstoffe ([14]; Tab. 1; [15]).

**Tab. 1:** Beispiele für aktive Immunisierungen gegen bakterielle Erreger [15, 61]

Impfung gegen	Erreger	Impfstoffart	Markenname
Pneumokokken	*Streptococcus pneumoniae*	Polysaccharid-Konjugate	Prevnar 13/20®, PNEUMOVAX 23®, Vaxneuvance®, Synflorix^TM^, CAPVAXIVE®, Apexxnar®
Lactobacillus (L.)	*L. rhamnosus, L. vaginalis, L. fermentum, L. salivarius*	8 inaktivierte Stämme	Gynatren®
Hib	*Haemophilus influenzae* Typ b	Polysaccharid-Konjugat	PedvaxHIB®, ActHIB®, HibTITER®
Pertussis	*Bordetella pertussis*	Toxoid	s. Kombinationsimpfstoff
Diphtherie	*Corneybacterium diphtheriae*	Toxoid	s. Kombinationsimpfstoff
Tetanus	*Clostridium tetani*	Toxoid	s. Kombinationsimpfstoff
Kombination aus Pertussis, Diphtherie, Tetanus	*Bordetella pertussis, Corneybacterium diphtheriae, Clostridium tetani*	Toxoide	Boostrix®, Infanrix®
Meningokokken C	*Neisseria meningitidis*	Polysaccharid-Konjugat	Menactra®, Menomune®, Menjugate®, NeisVac-C®
Meningokokken B	*Neisseria meningitidis*	2–4 Proteine	Bexsero®/4CMenB, Trumbena®
Meningokokken A, C, W, Y	*Neisseria meningitidis Gruppe A, C, W, Y*	4 Polysaccharid-Konjugate	Nimenrix®, Menveo, MenQuadfi®
Enterobakterien	*Escherichia coli, Morganella morganii, Proteus mirabilis, Klebsiella pneumoniae, Enterococcus faecalis*	Inaktivierte Stämme	StroVac®
Cholera	*Vibrio cholerae*	Choleratoxin‑B Untereinheit	Dukoral®
Typhus	*Salmonaella enterica *Typhi	Attenuierter Ty21-Stamm oder Polysaccharid-Konjugat	Typhoral L® Typhim Vi®

Ein klassisches Beispiel für einen inaktivierten Impfstoff ist der Ganzkeimtyphusimpfstoff, während der Bacillus-Calmette-Guérin-(BCG-)Impfstoff gegen Tuberkulose ein Beispiel für einen lebend attenuierten Impfstoff darstellt [16]. Diphtherie- und Tetanusimpfstoffe basieren auf chemisch inaktivierten Toxinen, während Pneumokokken- und Hib-Impfstoffe zu den Konjugatimpfstoffen zählen, bei denen Polysaccharide mit Trägerproteinen wie Diphtherietoxoid oder der nichttoxischen Mutante des Diphtherietoxins (Cross-Reactive Material 197, CRM197) verknüpft sind [17].

Die vor einem Vierteljahrhundert erreichte Fähigkeit, vollständige Genome von Bakterien zu sequenzieren, eröffnete auch neue Perspektiven für die Impfstoffentwicklung. Darunter die sogenannte reverse Vakzinologie, welche genomische Analysen nutzt, um *in silico* potenzielle Antigene zu identifizieren, die anschließend rekombinant exprimiert und in präklinischen Modellen getestet werden [18]. Ein prominentes Beispiel, das hierauf basiert, ist der Impfstoff Bexsero® (4CMenB), der insbesondere Säuglinge und Kleinkinder vor Meningokokken der Serogruppe B schützt [19].

In einem weiterführenden Ansatz (reverse Vakzinologie 2.0) werden Antikörper gegen zuvor ermittelte, protektive Antigene generiert und ebenfalls in Infektionsmodellen auf ihre Schutzwirkung untersucht. Die Antigen-Bindestellen (Epitope) der daraus resultierenden, protektiven Antikörper können dann identifiziert und ihrerseits als optimierte Impfstoffe in einem entsprechenden Format (z. B. als multivalenter oder polyvalenter Nanopartikel) zur aktiven Immunisierung eingesetzt werden [20, 21].

Darüber hinaus kommen strukturbiologische Methoden („strukturelle Vakzinologie“) zum Einsatz, um Epitope zu optimieren, die eine besonders protektive Immunantwort erzielen können, ohne allergische Reaktionen oder Reaktogenität hervorzurufen [22]. Zudem können Peptide von 20 bis 30 Aminosäuren kostengünstig synthetisiert und kombiniert werden, um gleichzeitig verschiedene Serotypen oder auch Zielproteine eines Pathogens abzudecken und damit eine effektivere und dauerhaftere Immunisierung zu erreichen. Trotz dieser offensichtlichen Vorteile befinden sich peptidbasierte Vakzine zumeist noch in der präklinischen Entwicklung [23] bzw. sind im Fall von *Streptococcus pyogenes* erst in der klinischen Phase I [24], denn sie beinhalten auch einige Nachteile. Peptidbasierte Impfstoffe besitzen zumeist nur eine schwache Immunogenität, verbunden mit einer eingeschränkten T‑Zell-Aktivierung durch unzureichende Antigenpräsentation und einer daraus resultierenden schwierigen Evaluierung im Tiermodell. Diese Nachteile können in Zukunft durch Verbesserung der Impfstoffkonstruktionstechnik mehr als ausgeglichen werden: Dazu gehören ein breites Antigenrepertoire, längere synthetische Peptide sowie die Einbindung von geeigneten Linkern und Trägerproteinen, Immunstimulanzien in Form von Helferpeptiden, Toll-like-Rezeptor-(TLR-)Agonisten und Adjuvanzien und der Einsatz transgener Tiermodelle mit humanen Histokompatibilitäts-Leukozyten-Antigen-(HLA-)Molekülen zur Evaluierung der Wirksamkeit [25].

Weitere Innovationen umfassen genetisch modifizierte Außenmembranvesikel von Bakterien [26], die als Trägerplattform für Antigene dienen, sowie die Entwicklung von Biokonjugaten in *Escherichia coli* zur kosteneffizienten Herstellung komplexer Impfstoffe [27].

Ebenfalls zu erwähnen ist die auf der mRNA-Technologie basierende Impfstoffentwicklung, die durch die COVID-19-Pandemie viel Aufmerksamkeit erlangt hat und zunehmend auch für bakterielle Pathogene untersucht wird. So konnten beispielsweise die Immunogenität und Wirksamkeit von selbst amplifizierenden mRNA-Impfstoffen, die Antigene von Streptokokken der Gruppen A (GAS, *Streptococcus pyogenes*) und B (GBS, *Streptococcus agalactiae*) exprimieren, in einem Maus-Infektionsmodell gezeigt werden [28].

### Wirkmechanismen

Impfungen gegen bakterielle Erreger initiieren eine mehrstufige Immunantwort, die mit der Aufnahme von Antigenen durch antigenpräsentierende Zellen beginnt (angeborene Immunantwort) und in eine adaptive Immunität übergeht, die humorale und zelluläre Komponenten umfasst, Gedächtnisbildung ermöglicht und langfristigen Schutz ausbildet. Nach der Verabreichung des Impfstoffs gelangen Antigene oder antigentragende Partikel in das Gewebe, wo lokale dendritische Zellen und Makrophagen das Material aufnehmen, in Peptide spalten und über Major-Histocompatibility-Complex-(MHC-)Klasse-II-Moleküle auf ihrer Oberfläche präsentieren. Cluster of Differentiation 4 (CD4^+^)-T-Helferzellen erkennen diese peptidbeladenen MHC-II-Moleküle über ihre jeweiligen T‑Zell-Rezeptoren und werden so gegebenenfalls aktiviert. Sie lassen sich ihren Aufgaben entsprechend in die Subtypen Th1, Th2 oder follikuläre T‑Helferzellen (Tfh) unterteilen, die spezifische T‑ oder B‑Zell-Reaktionen durch Zellkontakt und Zytokin-Ausschüttung unterstützen [29].

Prä-B-Zellen können über ihren B‑Zell-Rezeptor in Form eines membrangebundenen Immunglobulins M (IgM) ein entsprechend passendes Antigen binden, internalisieren und ebenfalls über MHC-Klasse-II-Moleküle auf ihrer Oberfläche präsentieren. Durch Interaktion mit einer antigenspezifischen Th2-Zelle werden die B‑Zellen aktiviert und wandern in die Keimzentren von Lymphfollikeln. Dort durchlaufen sie, unterstützt durch Tfh-Zellen, einen Klassenwechsel zu IgG, A oder E, eine Phase der somatischen Hypermutation und eine Affinitätsreifung durch Selektion zu Plasmazellen, die Antikörper mit der höchsten Affinität produzieren. Zusätzlich entstehen auch B‑Gedächtniszellen, die bei erneutem Kontakt mit dem jeweiligen Antigen eine schnelle humorale Immunreaktion ermöglichen und so beispielsweise bakterielle Endotoxine neutralisieren [30].

Je nach Erreger gibt es differente Immunpfade: Manche bakteriellen Erreger erfordern primär eine starke Humoralantwort, andere eine zelluläre Immunität oder eine Kombination beider Strategien. Die Eliminierung von intrazellulären Bakterien, wie z. B. *Salmonella Typhi, Listeria monocytogenes* oder *Mycobacterium tuberculosis,* erfolgt zumeist über Th1-Zellen. Diese aktivieren wiederum Makrophagen und zytotoxische CD8^+^-T-Zellen insbesondere durch Interferon‑γ (IFN-γ), Tumornekrosefaktor-alpha (TNF-α) und Interleukin 2 (IL-2) und fördern so die zelluläre Immunantwort. Bakterienspezifische Peptide werden von den infizierten Zellen über MHC-Klasse-I-Moleküle auf ihrer Oberfläche präsentiert und dadurch von den aktivierten, zytotoxischen CD8^+^-T-Zellen erkannt und eliminiert [31].

Extrazelluläre Bakterien werden dagegen primär über eine humorale Immunreaktion bekämpft, indem die dabei sezernierten Antikörper die Bakterien für phagozytierende Immunzellen oder das Komplementsystem markieren und dadurch ihre Effektivität steigern. Wiederholte Impulse, z. B. durch Auffrischimpfungen, festigen das Immungedächtnis durch Vergrößerung des Repertoires an B‑ und T‑Gedächtniszellen, erhöhen die Antikörperaffinität (Affinitätsreifung) und den Antikörpertiter im Serum [15].

Die Art des Impfstoffs beeinflusst die Ausprägung der Immunantwort. Lebend attenuierte Impfstoffe induzieren primär zelluläre Antworten, während Tot- oder Teilimpfstoffe eher Antikörperantworten stimulieren; Nukleinsäure-basierte Formulierungen können sowohl humorale als auch zelluläre Komponenten modulieren. Kulturelle und epidemiologische Faktoren, darunter das Alter, eine Impfakzeptanz, die immunologische Vorgeschichte und der individuelle Gesundheitszustand der zu impfenden Person, sowie der Impfstofftyp beeinflussen die Wirksamkeit und die Verträglichkeit [32]. Dies und die Wandlungsfähigkeit einiger bakterieller Pathogene erfordern Anpassungen der Impfstoffe und Auffrischungen der Impfungen.

### Adjuvanzien und Trägermoleküle

Adjuvanzien (Hilfsstoffe) verstärken die Immunantwort einer Impfung und damit auch die Langlebigkeit des Schutzes gegen bakterielle Erreger. Sie fördern die Antigenpräsentation sowie Antigenpersistenz („Depot-Effekt“) und induzieren dadurch sowohl eine verstärkte humorale Immunantwort als auch die zelluläre Immunantwort.

Klassische Beispiele von Adjuvanzien sind Aluminium-Derivate („Alum“), die seit den 1920er-Jahren eingesetzt werden. Moderne, beim Menschen zugelassene Adjuvanssysteme wie AS01 bestehen aus liposomalen Formulierungen, die MPL (monophosphoryliertes Lipid A) und ein Saponin (QS-21) kombinieren. MPL aktiviert über den TLR4 die antigenpräsentierenden Zellen, während QS-21 zusätzlich zytotoxische CD8⁺-T-Zell-Antworten und IL-1β‑/IL-18-Freisetzung über die Inflammasom-Aktivierung fördert [33]. Oligonukleotide wie CpG ODN, die unmethylierte bakterielle CpG-Sequenzen imitieren, dienen als synthetische TLR9-Agonisten. Diese aktivieren eine starke Th1-Immunantwort mit erhöhter zellvermittelter Immunität. CpG 7909 findet sich beispielsweise im Anthrax-Impfstoff Cyfendus [34]. Ein weiterer innovativer Ansatz sind TLR2-Agonisten, beispielsweise das synthetische Lipopeptid-Analogon Pam3Cys-SK‑4 (auch bekannt als Pam3CSK4). Dieser aktiviert über den TLR2/TLR1-Komplex starke, proinflammatorische Signale (z. B. NF-κB), fördert Phagozytose, Chemotaxis und bakterizide Aktivität insbesondere durch Neutrophile [35].

Trägermoleküle werden mit kleinen oder wenig immunogenen Antigenen, wie z. B. kurzen Peptiden oder Polysacchariden, fusioniert, damit diese auch von T‑Helferzellen erkannt werden und somit eine langlebige Immunität mit Gedächtniszellen ermöglicht wird. Bekannte Beispiele von Trägermolekülen sind das Tetanustoxoid und das Diphterietoxoid (CRM197), welche inaktivierte Gifte darstellen.

Insgesamt zeigt sich, dass der gezielte Einsatz verschiedener Adjuvanzien und Trägermoleküle die maßgeschneiderte Verstärkung von Immunantworten gegen bakterielle Pathogene ermöglicht, einschließlich Th1-, Th2- sowie CD8⁺-T-Zellen-Antwort und humoraler Reaktion. Ihre Kombination ermöglicht es, verbesserte Impfstoffe gegen anspruchsvolle, vor allem intrazelluläre Erreger zu entwickeln.

Mutmaßungen, dass insbesondere Aluminiumsalze verschiedene Erkrankungen hervorrufen könnten, darunter auch Autismus, wurden unter anderem durch eine Studie aus Dänemark aus dem Jahr 2025 widerlegt. Darin wurden die Daten von 1,2 Mio. Kindern bezüglich des Auftretens von 50 chronischen Erkrankungen nach Impfungen ausgewertet. Es konnte kein Zusammenhang zwischen Aluminiumgehalt des Impfstoffes und dem vermehrten Auftreten von neuronalen oder allergischen Reaktionen oder gar Autoimmunerkrankungen gezeigt werden [36].

Insgesamt ermöglicht die durch Impfung trainierte Immunantwort eine effektive Eindämmung bakterieller Infektionen durch eine koordinierte Aktivierung der angeborenen und adaptiven Immunreaktionen. Sie ist dabei abhängig von der Wahl des verwendeten Zielmoleküls, der Art des Impfstoffes und den gegebenenfalls zur Anwendung gekommenen Adjuvanzien.

## Passive Immunisierung

Die passive Immunisierung, definiert als die Verabreichung vorgefertigter Antikörper, stellt eine effektive Methode zur unmittelbaren Immunprophylaxe oder -therapie dar. Im Gegensatz zur aktiven Immunisierung induziert sie kein immunologisches Gedächtnis, bietet jedoch einen raschen Schutz, der insbesondere bei akut verlaufenden, Toxin-vermittelten Infektionen oder bei immunsupprimierten Patienten von entscheidender Bedeutung sein kann. Die zugrunde liegenden Wirkmechanismen umfassen die Neutralisation bakterieller Exotoxine, die Blockade der bakteriellen Adhärenz und Invasion, die Opsonisierung zur erleichterten Phagozytose durch Makrophagen und Granulozyten sowie die Aktivierung des Komplementsystems mit konsekutiver Lyse der Erreger (Tab. 2; [37]).

**Tab. 2:** Beispiele für passive Immunisierungen (verabreichte Antikörper) gegen bakterielle Erreger [41]

Erreger (Erkrankung)	Antikörperpräparat	Wirkmechanismus
*Clostridium tetani* (Tetanus)	Humanes Tetanus-Immunglobulin (TIG); Pferde-Antitoxin	Neutralisation von Tetanustoxin
*Clostridium botulinum* (Botulismus)	Humanes Botulismus-Immunglobulin (BIG-IV); Pferde-Antitoxin	Neutralisation von Botulinumtoxin
*Clostridioides difficile*	Bezlotoxumab	Bindung/Neutralisation von Toxin B
*Staphylococcus aureus* (Toxic-Shock-Syndrom)	Intravenöses Immunglobulin (IVIG); Pagibaximab (experimentell)	Neutralisation von Superantigenen; Opsonisierung
*Bacillus anthracis* (Milzbrand)	Raxibacumab, Anthrasil	Blockade des Schutzantigens (PA)
*Helicobacter pylori*	Orale bovine polyklonale Antikörper	Hemmung der Urease und Kolonisation

Historisch war die passive Immunisierung vor Einführung der Antibiotika die einzige wirksame Maßnahme zur Prävention und Behandlung bakterieller Erkrankungen wie Diphtherie, Tetanus, bakterieller Meningitis und Scharlach. In der modernen klinischen Praxis wird beispielsweise das humane Tetanus-Immunglobulin (TIG) als Postexpositionsprophylaxe bei unzureichend gegen Tetanus immunisierten Personen verabreicht; in Regionen ohne TIG steht equines Antitoxin zur Verfügung [38]. Beim Botulismus erfolgt die Therapie in Europa mit Antitoxin-Antikörperfragmenten gegen alle 7 bekannten Serotypen A–G (BAT), die aus Pferdeserum gewonnen werden [39].

Bei *Clostridioides-difficile*-Infektionen hat sich neben der Gabe von intravenösem Immunglobulin (IVIG) der monoklonale Antikörper Bezlotoxumab etabliert. Bezlotoxumab bindet spezifisch an Toxin B und konnte in randomisierten, kontrollierten Studien die Rezidivrate signifikant reduzieren, wenn er additiv zur Standardantibiotikatherapie eingesetzt wurde [40]. Im Rahmen des Staphylokokken-induzierten Toxic-Shock-Syndroms wird IVIG zur Neutralisation von Superantigenen und zur Modulation der überschießenden Zytokinantwort verabreicht [37].

Antikörperpräparate liegen entweder als polyklonale oder als monoklonale Produkte vor [41]. Polyklonale Antikörper werden meist aus humanem oder tierischem Serum gewonnen und decken ein breites Epitop-Spektrum ab. Sie unterliegen jedoch Chargenvariabilität und sind in der Herstellung auf geeignete Spenderpopulationen angewiesen. Monoklonale Antikörper wie Bezlotoxumab oder Raxibacumab (gegen das Schutzantigen von *Bacillus anthracis*) sind hochspezifisch und zeichnen sich durch reproduzierbare Qualität aus, sind jedoch kostenintensiv.

Antivirulenz-Strategien mit monoklonalen Antikörpern, beispielsweise Pagibaximab gegen die Lipoteichonsäure von *Staphylococcus (S.) aureus*, demonstrierten in präklinischen Studien vielversprechende Ergebnisse, konnten jedoch in klinischen Phase-III-Studien keine signifikanten Vorteile erzielen [37]. Ein weiteres innovatives Konzept stellen Lysibodies dar – gentechnisch konstruierte Antikörper, die Phagen-Domänen zur Erkennung konservierter bakterieller Zellwandstrukturen nutzen und so gezielt Komplementaktivierung und Phagozytose induzieren [42].

Limitationen der passiven Immunisierung ergeben sich aus der begrenzten Wirkdauer infolge des physiologischen Antikörperabbaus innerhalb weniger Wochen bis Monate, dem Risiko immunologischer Reaktionen, wie beispielsweise die Serumkrankheit insbesondere bei heterologen Seren, sowie den hohen Produktions- und Anwendungskosten, vor allem bei monoklonalen Antikörpern. Dennoch zeigt das Beispiel Bezlotoxumab, dass gezielt entwickelte monoklonale Antikörper die therapeutischen Optionen gegen spezifische, Toxin-vermittelte bakterielle Erkrankungen substanziell erweitern und insbesondere im Kontext steigender Antibiotikaresistenzen einen wachsenden Stellenwert einnehmen könnten.

## Epidemiologische Effekte von Impfungen gegen bakterielle Infektionen

Epidemiologische Daten belegen eindrucksvoll, dass Impfprogramme zur Reduktion antibiotikaresistenter Infektionen beitragen können. Die Einführung des Pneumokokken-Konjugatimpfstoffs führte zu einem deutlichen Rückgang invasiver Erkrankungen sowohl durch sensible als auch durch resistente Pneumokokkenstämme. Auch mit Hib-Impfprogrammen wurden nennenswerte Erfolge erzielt, die nicht nur die Krankheitslast reduzierten, sondern zusätzlich den Bedarf an empirischer Antibiotikatherapie bei Kindern senkten. Impfungen gegen Pertussis und Meningokokken trugen ebenfalls indirekt zur Verringerung des Antibiotikaverbrauchs bei, da sie Sekundärinfektionen und Krankheitsausbrüche verminderten [43]. Ähnliche Erfolge scheinen sich auch durch die Immunprophylaxe bei rezidivierenden Harnwegsinfektionen abzuzeichnen, indem die Verabreichung der häufigsten, inaktivierten Erreger wie *Escherichia coli, Morganella morganii, Proteus mirabilis, Klebsiella pneumoniae *und *Enterococcus faecalis* die Infektionshäufigkeit senkt und hilft, Antibiotikaresistenzen zu vermeiden [44].

Neben dem individuellen Schutz spielt die Herdenimmunität eine zentrale Rolle. Sie reduziert die Zirkulation des Erregers in der Bevölkerung, wodurch auch nicht geimpfte Personen indirekt geschützt werden [45]. In diesem Zusammenhang kann auch der Nestschutz aufgeführt werden: Durch Impfung während der Schwangerschaft werden die generierten Antikörper über die Plazenta auf das Ungeborene bzw. durch das Stillen auf das Neugeborene übertragen und schützen es nach der Geburt in den ersten Lebensmonaten vor möglichen Infektionen. Die Ständige Impfkommission (STIKO) empfiehlt seit 2020 eine Impfung gegen Pertussis während der Schwangerschaft, um damit das Risiko einer möglichen Infektion um 90 % zu senken [46].

Im Gegensatz zu Antibiotika verändern Impfstoffe nicht die Zusammensetzung des intestinalen Mikrobioms, das eine wichtige Barriere gegen invasive Pathogene darstellt [47]. Antibiotikatherapien können dagegen zu einer Dysbiose führen, die opportunistischen Erregern wie *Clostridioides difficile* oder multiresistenten Enterobakterien einen selektiven Vorteil verschafft [48].

Für die Prävention von AMR sind Impfstoffe aus den folgenden 3 Gründen besonders geeignet: Erstens werden sie in der Regel prophylaktisch verabreicht, wenn die Erregerpopulation im Körper noch gering oder nicht vorhanden ist. Dies minimiert die Wahrscheinlichkeit, dass resistente Mutationen entstehen und sich etablieren können [49]. Zweitens zielen viele Impfstoffe auf mehrere Antigenstrukturen gleichzeitig, was die Wahrscheinlichkeit einer Resistenzentwicklung erheblich reduziert, da dafür mehrere genetische Veränderungen am Zielprotein notwendig wären. Drittens aktiviert die Impfung viele Teile des Immunsystems (humorale Antwort, Gedächtniszellen, zelluläre Immunität), sodass es bei einer Infektion dem Erreger nur selten gelingt, die Immunantwort durch „Immunescape“-Strategien zu umgehen. Ein Beispiel für so einen seltenen Fall ist *S. aureus*, gegen den bisher noch kein Vakzin verfügbar ist, da dieser Erreger besonders viele Virulenzfaktoren exprimiert [50].

## Ökonomische und gesundheitspolitische Aspekte von Impfprogrammen

Neben gesundheitlichen Vorteilen bieten Impfprogramme auch ökonomische Anreize für die Volkswirtschaft. Modellrechnungen zufolge können Investitionen in Impfstoffe gegen bakterielle Pathogene kosteneffizienter sein als die ausschließliche Entwicklung neuer Antibiotika [51]. Insbesondere bei Risikopatienten (vor Operationen, Gesundheitspersonal, Soldaten im Kriegseinsatz, immunsupprimierte Patienten) werden Impfungen als sinnvoll erachtet. Die Einsparungen resultieren aus der Vermeidung von Krankenhausaufenthalten, der Reduktion von Behandlungskosten für resistente Infektionen und der Verringerung der Umweltbelastungen bei der Produktion von Antibiotika. Besonders in Ländern mit eingeschränkter medizinischer Infrastruktur, in denen Antibiotika oft ohne ärztliche Verschreibung verabreicht werden, könnten Impfprogramme eine zentrale Rolle bei der Eindämmung von AMR spielen [52].

Trotz ihres Potenzials stehen Impfprogramme vor vielfältigen Herausforderungen. Gegen einige der von der Weltgesundheitsorganisation (WHO) priorisierten bakteriellen Erreger, wie *Klebsiella pneumoniae* oder *Acinetobacter baumannii*, existieren bisher keine zugelassenen Impfstoffe [53]. Zudem können zum Beispiel sehr seltene Impfkomplikationen, die durch das sensible Meldesystem erkannt werden, die öffentliche Wahrnehmung von Impfungen negativ beeinflussen. Impfgegnerbewegungen und Desinformationskampagnen unterminieren in manchen Regionen die Impfbereitschaft und können politische Entscheidungen beeinflussen [54]. Dies ist ein nicht zu unterschätzender Faktor, denn der Erfolg von Impfkampagnen hängt ganz wesentlich von der Akzeptanz in der Bevölkerung ab, wenn Impfungen nicht gesetzlich vorgeschrieben sind. Die Faktenlage zeigt allerdings, dass beispielsweise bei insgesamt 105 Mio. Impfungen nur 8659 Verdachtsfälle von Impfnebenwirkungen bzw. Impfkomplikationen in den Jahren 2022 bis 2023 gemeldet wurden (Fieber, Kopfschmerzen, Fieberkrämpfe, Epilepsie, Guillain-Barré-Syndrom etc.). Das entspricht einem Anteil von 0,008 %, wobei allerdings in den meisten Fällen kein gesichert kausaler Zusammenhang festgestellt werden kann [55].

Der Erfolg einer Impfkampagne hängt nicht nur von der Akzeptanz und Wirksamkeit des Impfstoffs ab, sondern auch von der Zugänglichkeit des Impfstoffs. Bedingt durch finanzielle, logistische und gesellschaftspolitische Faktoren können eine gleichmäßige Verteilung von Impfstoffen und damit sowohl der Aufbau des individuellen Schutzes als auch der angestrebten Herdenimmunität deutlich erschwert werden [56].

Für die Pharmaindustrie ist neben den Entwicklungskosten und dem Verkaufspreis das Marktpotenzial eines Medikaments entscheidend. Seit 2011 sind 11 neue Antibiotika zugelassen worden. Einige von diesen (z. B. Tedizolid, Eravacyclin) dürfen allerdings aufgrund ihrer Wirksamkeit gegen multiresistente Bakterien als sogenannte Reserveantibiotika nur unter bestimmten Bedingungen verabreicht werden, sodass sich damit kaum Geld verdienen lässt [57]. Impfstoffe hingegen haben zwar auch hohe Entwicklungs- und Produktionskosten, können aber nach Marktzulassung als prophylaktisches Medikament uneingeschränkt und langfristig verkauft werden, wie beispielsweise der Kombinationsimpfstoff gegen Tetanus, Diphtherie und Pertussis.

## Entwicklung neuer Impfstoffe

Die Entwicklung neuer Impfstoffe wird trotz der geschilderten Schwierigkeiten von der Pharmaindustrie und von Forschungsinstituten weiterverfolgt, da diese anerkanntermaßen einen wichtigen Pfeiler im Kampf gegen bakterielle Infektionen bilden. Dabei ist insbesondere die Impfung gegen obligat intrazelluläre Bakterien aufgrund spezifischer Immunanforderungen zur Abwehr und zum Schutz durch die Aktivierung zytotoxischer CD8^+^-T-Zellen eine Herausforderung. Es stehen mittlerweile mehrere Impfstrategien der dritten Generation zur Verfügung, die vielversprechende Instrumente für die Impfung auch gegen diese Erreger darstellen könnten.

Die schützenden Immunmechanismen gegen verschiedene bakterielle Erreger sind jedoch noch wenig verstanden und bedürfen weiterer Forschung. Insbesondere die immunogenen Determinanten dieser komplexen Erreger sind weitgehend unbekannt und müssen dringend als Voraussetzung für die Impfstoffentwicklung identifiziert werden. Dies erfordert geeignete Tiermodelle, die der menschlichen Infektion ähneln, und die Auswahl geeigneter Adjuvanzien, die entscheidend für den Erfolg der Immunisierung sind.

Ausgehend von der Prävalenz von multiresistenten Bakterien hat die WHO 2024 eine Prioritätenliste für die Antibiotikaentwicklung erstellt, an der sich auch die Impfstoffentwicklung orientiert. Demnach sind alternative Behandlungsmöglichkeiten gegen *Acinetobacter baumannii, Pseudomonas aeruginosa, Enterobacterales spp., Enterococcus faecium, S. aureus, Salmonella spp., Neisseria gonorrhoeae, Streptococcus spp., Haemophilus influenzae, Mycobacterium tuberculosis und *Shigella spp. dringend erforderlich [53]. Die Tab. 3 und 4 geben einen Überblick über die momentanen Impfstoffentwicklungen [58].

**Tab. 3:** In der Entwicklung befindliche passive Immunisierungen gegen bakterielle Erreger (Stand: 11/2022). Die Tabellen beruhen auf Recherchen des Verbands der forschenden Pharma-Unternehmen (vfa) unter Verwendung von Veröffentlichungen der genannten Unternehmen, PharmaProjects, Angaben der Europäischen Arzneimittel-Agentur (EMA) und der Studienregister clinicaltrials.gov.

Name des Antikörpers	Klasse	Unternehmen	Anwendungsgebiete	Entwicklungsstatus
Obiltoxaximab	Monoklonaler Antikörper	Elysys Therapeutics	Milzbrand-Infektionen	EU-Zulassung 11/2020, aber in Deutschland noch nicht vermarktet
Aerumab (Panobacumab, AR-101)	Monoklonaler Antikörper	Aridis Pharmaceuticals	*Pseudomonas-aeruginosa*-Infektionen	Phase II
Tosatoxumab (AR-301, Salvecin)	Monoklonaler Antikörper	Aridis Pharmaceuticals	Infektionen mit *Staphylococcus aureus,* einschl. MRSA	Phase III
Suvratoxumab (Medi-4893)	Monoklonaler Antikörper gegen Toxin	Aridis Pharmaceuticals	MRSA-Infektionen	Phase II
LMN-101	Antikörper-ähnliches Protein	Lumen Bioscience	Infektionen mit *Escherichia coli* oder *Campylobacter jejuni*	Phase II

*MRSA* Methicillin-resistente *Staphylococcus *aureus

**Tab. 4:** In der Entwicklung befindliche aktive Immunisierungen gegen bakterielle Erreger (Stand: 11/2022). Die Tabellen beruhen auf Recherchen des Verbands der forschenden Pharma-Unternehmen (vfa) unter Verwendung von Veröffentlichungen der genannten Unternehmen, PharmaProjects, Angaben der Europäischen Arzneimittel-Agentur (EMA) und der Studienregister clinicaltrials.gov.

Name des Impfstoffprojektes	Impfstofftyp	Unternehmen, Organisation	Anwendungsgebiet	Entwicklungsstatus
PF-06425090	Subunit-Impfstoff	Pfizer	Prävention von *Clostridioides-difficile*-Infektionen	Phase III
ExPeC9V	Konjugatimpfstoff	Janssen	Prävention von *Escherichia-Coli*-Infektionen	Phase III
GSK3536819A	Impfstoff	GSK	Infektionen mit Meningokokken der Serogruppen A, B, C, W und Y	Phase III
PF-06886992	Konjugatimpfstoff	Pfizer	Infektionen mit Meningokokken der Serogruppen A, B, C, W und Y	Phase III
PF-06760805	Nicht angegeben	Pfizer	Prävention von Infektionen bei Neugeborenen mit Streptokokken der Gruppe B durch Impfung der Mütter	Phase II
VLA84 (IC84)	Subunit-Impfstoff	Valneva	Prävention von *Clostridioides-difficile*-Infektionen	Phase II
VPM 1002	Lebendimpfstoff, attenuiert, rekombinant	Serum Institute of India, Max Planck, VPM	Prävention und Therapie von Tuberkulose	Phase III
*Mycobacterium vaccae*	Subunit-Impfstoff	Anhui Zhifei Longcom	Prävention von Tuberkulose	Phase III
GC-3107	Intradermaler BCG-Impfstoff	GC-Pharma	Tuberkulose	Phase III
MTBVac	Lebendimpfstoff	Biofabri, Univ. Zagaroza, TBVI	Prävention von Tuberkulose	Phase II
TBFluO4L	Impfstoff mit Vektor	RIBSP	Prävention und Therapie von Tuberkulose	Phase II
RUTI	Ganzzellimpfstoff	Archivel Farma	Prävention und Therapie von Tuberkulose	Phase II
PF-07307405	Impfstoff	Pfizer, Valneva	Lyme-Borreliose	Phase II
Shigella4V (GSK4069327A)	Konjugatimpfstoff	GSK	Shigella-Infektionen	Phase II
ASP3772	Konjugatimpfstoff	Affinivax, Astellas	Infektionen mit *Streptococcus pneumoniae* (24 Serogruppen)	Phase II
V116	Konjugatimpfstoff	MSD	Infektionen mit *Streptococcus pneumoniae*	Phase I/II

## Fazit

Zusammenfassend stellen Impfstoffe eine der nachhaltigsten und wirksamsten Maßnahmen zur Prävention bakterieller Infektionen und zur Eindämmung von Antibiotikaresistenzen dar. Durch die Senkung der Infektionsrate und des Antibiotikaverbrauchs reduzieren sie den Selektionsdruck auf Bakterien und verlangsamen die Ausbreitung resistenter Stämme. Angesichts der stagnierenden Entwicklung neuer Antibiotika sollten Impfstrategien integraler Bestandteil nationaler und globaler AMR-Bekämpfungsprogramme sein. Investitionen in Forschung, Entwicklung und Implementierung neuer Impfstoffe sowie in Aufklärungsprogramme sind entscheidend, um das volle Potenzial dieser Maßnahme im Kampf gegen die Resistenzkrise auszuschöpfen [59, 60].

## References

[1] Fleming A (1929) On the antibacterial action of cultures of a penicillium, with special reference to their use in the isolation of B. influenzae. Br J Exp Pathol 10:226–236 PMC2566493 11545337

[2] Holmes AH, Moore LSP, Sundsfjord A et al (2016) Understanding the mechanisms and drivers of antimicrobial resistance. Lancet Lond Engl 387:176–187. 10.1016/S0140-6736(15)00473-0 10.1016/S0140-6736(15)00473-0 26603922

[3] O’Neill J (2016) Tackling drug-resistant infections globally: Final report and recommendations. https://amr-review.org/sites/default/files/160518_Final%20paper_with%20cover.pdf. Accessed 20.1.2026

[4] GBD 2021 Antimicrobial Resistance Collaborators (2024) Global burden of bacterial antimicrobial resistance 1990-2021: a systematic analysis with forecasts to 2050. Lancet 404:1199–1226. 10.1016/S0140-6736(24)01867-1 39299261 10.1016/S0140-6736(24)01867-1 PMC11718157

[5] Van Boeckel TP, Brower C, Gilbert M et al (2015) Global trends in antimicrobial use in food animals. Proc Natl Acad Sci U S A 112:5649–5654. 10.1073/pnas.1503141112 25792457 10.1073/pnas.1503141112 PMC4426470

[6] Heimann D, Kohnhäuser D, Kohnhäuser AJ, Brönstrup M (2025) Antibacterials with Novel Chemical Scaffolds in Clinical Development. Drugs 85:293–323. 10.1007/s40265-024-02137-x 39847315 10.1007/s40265-024-02137-x PMC11891108

[7] Dyar OJ, Huttner B, Schouten J, Pulcini C, ESGAP (ESCMID Study Group for Antimicrobial stewardshiP) (2017) What is antimicrobial stewardship? Clin Microbiol Infect Off Publ Eur Soc Clin Microbiol Infect Dis 23:793–798. 10.1016/j.cmi.2017.08.026 10.1016/j.cmi.2017.08.026 28882725

[8] Klugman KP, Black S (2018) Impact of existing vaccines in reducing antibiotic resistance: Primary and secondary effects. Proc Natl Acad Sci U S A 115:12896–12901. 10.1073/pnas.1721095115 30559195 10.1073/pnas.1721095115 PMC6304973

[9] Lipsitch M, Siber GR (2016) How Can Vaccines Contribute to Solving the Antimicrobial Resistance Problem? mBio 7:e00428-16. 10.1128/mBio.00428-16 27273824 10.1128/mBio.00428-16 PMC4959668

[10] Jenner E (1959) An inquiry into the causes and effects of the variolae vaccinae: a disease discovered in some of the western counties of England, particularly Gloucestershire, and known by the name of the cow pox. London, 1798. Class Med Surg Ed Camac CNB:213–40

[11] Pasteur L, Chamberland CE, Roux E (1881) Sur la vaccination charbonneuse. CR Acad SciParis:1378–1383

[12] Plotkin SA (2005) Vaccines: past, present and future. Nat Med 11:S5–11. 10.1038/nm1209 15812490 10.1038/nm1209 PMC7095920

[13] Peltola H (2000) Worldwide Haemophilus influenzae type b disease at the beginning of the 21st century: global analysis of the disease burden 25 years after the use of the polysaccharide vaccine and a decade after the advent of conjugates. Clin Microbiol Rev 13:302–317. 10.1128/CMR.13.2.302 10756001 10.1128/cmr.13.2.302-317.2000 PMC100154

[14] Poolman JT (2020) Expanding the role of bacterial vaccines into life-course vaccination strategies and prevention of antimicrobial-resistant infections. NPJ Vaccines 5:84. 10.1038/s41541-020-00232-0 32963814 10.1038/s41541-020-00232-0 PMC7486369

[15] Osterloh A (2022) Vaccination against Bacterial Infections: Challenges, Progress, and New Approaches with a Focus on Intracellular Bacteria. Vaccines 10:751. 10.3390/vaccines10050751 35632507 10.3390/vaccines10050751 PMC9144739

[16] Ritz N, Curtis N (2009) Mapping the global use of different BCG vaccine strains. Tuberc Edinb Scotl 89:248–251. 10.1016/j.tube.2009.03.002 10.1016/j.tube.2009.03.002 19540166

[17] Ul Haq MZ, Irfan H, Shah MSU et al (2025) Immunogenicity, safety and tolerability of 15-valent pneumococcal conjugate vaccine (V114) compared to 13-valent pneumococcal conjugate vaccine (PCV-13) in healthy infants: A systematic review and meta-analysis. Vaccine X 24:100654. 10.1016/j.jvacx.2025.100654

[18] Khalid K, Poh CL (2023) The Promising Potential of Reverse Vaccinology-Based Next-Generation Vaccine Development over Conventional Vaccines against Antibiotic-Resistant Bacteria. Vaccines 11:1264. 10.3390/vaccines11071264 37515079 10.3390/vaccines11071264 PMC10385262

[19] Abitbol V, Martinón-Torres F, Taha MK et al (2024) 4CMenB journey to the 10-year anniversary and beyond. Hum Vaccines Immunother 20:2357924. 10.1080/21645515.2024.2357924 10.1080/21645515.2024.2357924 PMC11232649 38976659

[20] Klimka A, Mertins S, Nicolai AK et al (2021) Epitope-specific immunity against Staphylococcus aureus coproporphyrinogen III oxidase. NPJ Vaccines 6:11. 10.1038/s41541-020-00268-2 33462229 10.1038/s41541-020-00268-2 PMC7813823

[21] Rappuoli R, Bottomley MJ, D’Oro U, Finco O, De Gregorio E (2016) Reverse vaccinology 2.0: Human immunology instructs vaccine antigen design. J Exp Med 213:469–481. 10.1084/jem.20151960 27022144 10.1084/jem.20151960 PMC4821650

[22] Sesterhenn F, Bonet J, Correia BE (2018) Structure-based immunogen design — leading the way to the new age of precision vaccines. Curr Opin Struct Biol 51:163–169. 10.1016/j.sbi.2018.06.002 29980105 10.1016/j.sbi.2018.06.002 PMC7127059

[23] Gong W, Pan C, Cheng P, Wang J, Zhao G, Wu X (2022) Peptide-Based Vaccines for Tuberculosis. Front Immunol 13:830497. 10.3389/fimmu.2022.830497 35173740 10.3389/fimmu.2022.830497 PMC8841753

[24] Meier-Stephenson V, Hawkes MT, Burton C et al (2024) A phase 1 randomized controlled trial of a peptide-based group A streptococcal vaccine in healthy volunteers. Trials 25:781. 10.1186/s13063-024-08634-4 39563457 10.1186/s13063-024-08634-4 PMC11577953

[25] Lloren KKS, Senevirathne A, Lee JH (2024) Advancing vaccine technology through the manipulation of pathogenic and commensal bacteria. Mater Today Bio 29:101349. 10.1016/j.mtbio.2024.101349 39850273 10.1016/j.mtbio.2024.101349 PMC11754135

[26] Behrens F, Funk-Hilsdorf TC, Kuebler WM, Simmons S (2021) Bacterial Membrane Vesicles in Pneumonia: From Mediators of Virulence to Innovative Vaccine Candidates. Int J Mol Sci 22:3858. 10.3390/ijms22083858 33917862 10.3390/ijms22083858 PMC8068278

[27] Kay E, Cuccui J, Wren BW (2019) Recent advances in the production of recombinant glycoconjugate vaccines. Npj Vaccines 4:16. 10.1038/s41541-019-0110-z 31069118 10.1038/s41541-019-0110-z PMC6494827

[28] Maruggi G, Chiarot E, Giovani C et al (2017) Immunogenicity and protective efficacy induced by self-amplifying mRNA vaccines encoding bacterial antigens. Vaccine 35:361–368. 10.1016/j.vaccine.2016.11.040 27939014 10.1016/j.vaccine.2016.11.040

[29] Chi H, Pepper M, Thomas PG (2024) Principles and therapeutic applications of adaptive immunity. Cell 187:2052–2078. 10.1016/j.cell.2024.03.037 38670065 10.1016/j.cell.2024.03.037 PMC11177542

[30] McConnell SA, Casadevall A (2025) New insights into antibody structure with implications for specificity, variable region restriction and isotype choice. Nat Rev Immunol 25:621–632. 10.1038/s41577-025-01150-9 40113994 10.1038/s41577-025-01150-9

[31] Tian L, Zhou W, Wu X et al (2022) CTLs: Killers of intracellular bacteria. Front Cell Infect Microbiol 12:967679. 10.3389/fcimb.2022.967679 36389159 10.3389/fcimb.2022.967679 PMC9645434

[32] Ghattas M, Dwivedi G, Lavertu M, Alameh MG (2021) Vaccine Technologies and Platforms for Infectious Diseases: Current Progress, Challenges, and Opportunities. Vaccines 9:1490. 10.3390/vaccines9121490 34960236 10.3390/vaccines9121490 PMC8708925

[33] Roman F, Burny W, Ceregido MA et al (2024) Adjuvant system AS01: from mode of action to effective vaccines. Expert Rev Vaccines 23:715–729. 10.1080/14760584.2024.2382725 39042099 10.1080/14760584.2024.2382725

[34] Goetz M, Thotathil N, Zhao Z, Mitragotri S (2024) Vaccine adjuvants for infectious disease in the clinic. Bioeng Transl Med 9:e10663. 10.1002/btm2.10663 39036089 10.1002/btm2.10663 PMC11256182

[35] Chen Y, Lu S, Zhang Y et al (2019) TLR2 agonist Pam3CSK4 enhances the antibacterial functions of GM-CSF induced neutrophils to methicillin-resistant Staphylococcus aureus. Microb Pathog 130:204–212. 10.1016/j.micpath.2019.02.030 30885749 10.1016/j.micpath.2019.02.030

[36] Andersson NW, Bech Svalgaard I, Hoffmann SS, Hviid A (2025) Aluminum-Adsorbed Vaccines and Chronic Diseases in Childhood : A Nationwide Cohort Study. Ann Intern Med 178:1369–1377. 10.7326/ANNALS-25-00997 40658954 10.7326/ANNALS-25-00997

[37] DiGiandomenico A, Sellman BR (2015) Antibacterial monoclonal antibodies: the next generation? Curr Opin Microbiol 27:78–85. 10.1016/j.mib.2015.07.014 26302478 10.1016/j.mib.2015.07.014

[38] Kabura L, Ilibagiza D, Menten J, Van den Ende J (2006) Intrathecal vs. intramuscular administration of human antitetanus immunoglobulin or equine tetanus antitoxin in the treatment of tetanus: a meta-analysis. Trop Med Int Health TM IH 11:1075–1081. 10.1111/j.1365-3156.2006.01659.x 16827708 10.1111/j.1365-3156.2006.01659.x

[39] Parrera GS, Astacio H, Tunga P, Anderson DM, Hall CL, Richardson JS (2021) Use of Botulism Antitoxin Heptavalent (A, B, C, D, E, F, G)-(Equine) (BAT®) in Clinical Study Subjects and Patients: A 15-Year Systematic Safety Review. Toxins 14:19. 10.3390/toxins14010019 35050996 10.3390/toxins14010019 PMC8778610

[40] Wilcox MH, Gerding DN, Poxton IR et al (2017) Bezlotoxumab for Prevention of Recurrent Clostridium difficile Infection. N Engl J Med 376:305–317. 10.1056/NEJMoa1602615 28121498 10.1056/NEJMoa1602615

[41] Prygiel M, Mosiej E, Wdowiak K, Zasada AA (2024) Passive Immunisation in the Treatment of Infectious Diseases Related to Highly Potent Bacterial Toxins. Biomedicines 12:2920. 10.3390/biomedicines12122920 39767826 10.3390/biomedicines12122920 PMC11673946

[42] Raz A, Serrano A, Lawson C et al (2017) Lysibodies are IgG Fc fusions with lysin binding domains targeting Staphylococcus aureus wall carbohydrates for effective phagocytosis. Proc Natl Acad Sci U S A 114:4781–4786. 10.1073/pnas.1619249114 28428342 10.1073/pnas.1619249114 PMC5422811

[43] World Health Organization (2024) Estimating the impact of vaccines in reducing antimicrobial resistance and antibiotic use. https://iris.who.int/server/api/core/bitstreams/b3bfc77f-c8fb-4076-a0e8-3b50780925ee/content. Accessed 23.1.2026

[44] Nestler S, Grüne B, Schilchegger L, Suna A, Perez A, Neisius A (2021) Efficacy of vaccination with StroVac for recurrent urinary tract infections in women: a comparative single-centre study. Int Urol Nephrol 53:2267–2272. 10.1007/s11255-021-02987-4 34499326 10.1007/s11255-021-02987-4

[45] Fine P, Eames K, Heymann DL (2011) „Herd immunity“: a rough guide. Clin Infect Dis Off Publ Infect Dis Soc Am 52:911–916. 10.1093/cid/cir007 10.1093/cid/cir007 21427399

[46] Vygen-Bonnet S, Hellenbrand W, Garbe E et al (2020) Safety and effectiveness of acellular pertussis vaccination during pregnancy: a systematic review. BMC Infect Dis 20:136. 10.1186/s12879-020-4824-3 32054444 10.1186/s12879-020-4824-3 PMC7020352

[47] Tedijanto C, Olesen SW, Grad YH, Lipsitch M (2018) Estimating the proportion of bystander selection for antibiotic resistance among potentially pathogenic bacterial flora. Proc Natl Acad Sci U S A 115:E11988–E11995. 10.1073/pnas.1810840115 30559213 10.1073/pnas.1810840115 PMC6304942

[48] Becattini S, Taur Y, Pamer EG (2016) Antibiotic-Induced Changes in the Intestinal Microbiota and Disease. Trends Mol Med 22:458–478. 10.1016/j.molmed.2016.04.003 27178527 10.1016/j.molmed.2016.04.003 PMC4885777

[49] Jansen KU, Gruber WC, Simon R, Wassil J, Anderson AS (2021) The impact of human vaccines on bacterial antimicrobial resistance. A review. Environ Chem Lett 19:4031–4062. 10.1007/s10311-021-01274-z 34602924 10.1007/s10311-021-01274-z PMC8479502

[50] Goldmann O, Medina E (2018) Staphylococcus aureus strategies to evade the host acquired immune response. Int J Med Microbiol IJMM 308:625–630. 10.1016/j.ijmm.2017.09.013 28939437 10.1016/j.ijmm.2017.09.013

[51] Sevilla JP, Bloom DE, Cadarette D, Jit M, Lipsitch M (2018) Toward economic evaluation of the value of vaccines and other health technologies in addressing AMR. Proc Natl Acad Sci U S A 115:12911–12919. 10.1073/pnas.1717161115 30559203 10.1073/pnas.1717161115 PMC6305008

[52] Akegbe H, Onyeaka H, et MM et al (2023) The need for Africa to develop capacity for vaccinology as a means of curbing antimicrobial resistance. Vaccine X 14:100320. 10.1016/j.jvacx.2023.100320 37293248 10.1016/j.jvacx.2023.100320 PMC10244683

[53] World Health Organization (2024) WHO Bacterial Priority Pathogens List, 2024. https://iris.who.int/server/api/core/bitstreams/1a41ef7e-dd24-4ce6-a9a6-1573562e7f37/content. Accessed 13.1.2026

[54] Larson HJ, Jarrett C, Eckersberger E, Smith DMD, Paterson P (2014) Understanding vaccine hesitancy around vaccines and vaccination from a global perspective: a systematic review of published literature, 2007–2012. Vaccine 32:2150–2159. 10.1016/j.vaccine.2014.01.081 24598724 10.1016/j.vaccine.2014.01.081

[55] Bundesinstitut für Arzneimittel und Medizinprodukte, Paul-Ehrlich-Institut (2024) Bulletin zur Arzneimittelsicherheit, Ausgabe 3. https://www.pei.de/SharedDocs/Downloads/DE/newsroom/bulletin-arzneimittelsicherheit/2024/3-2024.html. Accessed 30.9.2025

[56] Spooner F, Dattani S, Vanderslott S, Roser M (2022) Vaccination. https://ourworldindata.org/vaccination. Accessed 2.10.2025

[57] Verband Forschender Arzneimittelhersteller e. V. Antibiotika: Bestandsaufnahme zu Präparaten und Unternehmen. https://www.vfa.de/de/forschung-entwicklung/antibiotika/neue-antibiotika. Zugegriffen: 13. Januar 2026

[58] Perrie Y, Kaur R, Henriksen-Lacey M (2013) Vaccines. In: Uchegbu IF, Schätzlein AG, Cheng WP, Lalatsa A (eds) Fundam. Pharm. Nanosci. Springer New York, New York, NY, pp 465–491

[59] Rappuoli R, Bloom DE, Black S (2017) Deploy vaccines to fight superbugs. Nature 552:165–167. 10.1038/d41586-017-08323-0 29239371 10.1038/d41586-017-08323-0

[60] Wellcome Trust, International Federation of Pharmaceutical Manufacturers Associations (2016) Vaccines to tackle drug-resistant infections: An evaluation of R opportunities. https://vaccinesforamr.org/wp-content/uploads/2018/09/Vaccines_for_AMR.pdf. Accessed 30.9.2025

[61] Rote Liste® Service GmbH Bakterielle Impfstoffe - Hauptgruppenverzeichnis. https://www.rote-liste.de/hauptgruppenverzeichnis/75.3.1./Bakterielle-Impfstoffe. Zugegriffen: 15. Januar 2026

